# Mechanism of *SCD* Participation in Lipid Droplet-Mediated Steroidogenesis in Goose Granulosa Cells

**DOI:** 10.3390/genes13091516

**Published:** 2022-08-24

**Authors:** Xin Yuan, Ibn Iddriss Abdul-Rahman, Shenqiang Hu, Liang Li, Hua He, Lu Xia, Jiwei Hu, Mingxia Ran, Yali Liu, Mariama Abdulai, Jiwen Wang

**Affiliations:** 1 Country Farm Animal Genetic Resources Exploration and Innovation Key Laboratory of Sichuan Province, College of Animal Science and Technology, Sichuan Agricultural University, Chengdu 611130, China; 2 Department of Veterinary Science, Faculty of Agriculture, University for Development Studies, Nyankpala Campus, Tamale P.O. Box TL 1882, Ghana

**Keywords:** *SCD*, goose granulosa cells, lipid droplets, steroidogenesis

## Abstract

Stearoyl-CoA desaturase (*SCD*) is a key enzyme catalyzing the rate-limiting step in monounsaturated fatty acids (MUFAs) production. There may be a mechanism by which *SCD* is involved in lipid metabolism, which is assumed to be essential for goose follicular development. For this reason, a cellular model of *SCD* function in goose granulosa cells (GCs) via *SCD* overexpression and knockdown was used to determine the role of *SCD* in GC proliferation using flow cytometry. We found that *SCD* overexpression induced and *SCD* knockdown inhibited GCs proliferation. Furthermore, ELISA analysis showed that *SCD* overexpression increased the total cholesterol (TC), progesterone, and estrogen levels in GCs, while *SCD* knockdown decreased TC, progesterone, and estrogen levels (*p* < 0.05). Combining these results with those of related multi-omics reports, we proposed a mechanism of *SCD* regulating the key lipids and differentially expressed gene (DEGs) in glycerophospholipid and glycerolipid metabolism, which participate in steroidogenesis mediated by the lipid droplet deposition in goose GCs. These results add further insights into understanding the lipid metabolism mechanism of goose GCs.

## 1. Introduction

Recent research has revealed the significant role of stearoyl-CoA desaturase (*SCD*) in lipid metabolism in vivo [1,2]. *SCD* is a pivotal lipogenic enzyme predominantly located in the endoplasmic reticulum (ER). It converts saturated long-chain fatty acids (SFAs) into monounsaturated fatty acids (MUFAs) and is the rate-limiting enzyme in MUFA biosynthesis in vivo [3]. The resulting MUFAs are major components of triglycerides, cholesterol esters, and phospholipids [4,5,6,7]. In addition to being lipid components, MUFAs may also serve as mediators of signal transduction, cellular differentiation, and apoptosis [8,9]. Alteration in *SCD* expression alters the fatty acid profile of these lipids, yielding diverse effects on cellular function. High *SCD* activity and alteration in the balance between saturated and monounsaturated fatty acids are implicated in various diseases, including cancer, diabetes, atherosclerosis, and obesity [8,10].

Lipid droplets (LDs) are fat-storing organelles comprising a hydrophobic core of triacylglycerols (TAGs) and cholesterol esters surrounded by a phospholipid monolayer with various embedded proteins [11,12]. Accumulation of LDs in the cytoplasm plays a vital role in energy balance, membrane synthesis, and cell signal transduction. The turnover of LDs (e.g., through hydrolysis) can transfer hormone synthesis precursors, release lipid components for membrane synthesis, and secrete signaling molecules as lipid mediators [13]. Steroidogenic cells of the adrenal cortex, testes, and ovaries contain tiny LDs that primarily store cholesterol esters and serve as a reservoir of cholesterol for the synthesis and maintenance of membranes [14,15]. Although all animal cells package and store neutral lipids as discrete intracellular storage droplets, there is little information on the molecular processes that govern the deposition or catabolism of stored lipid components.

Indigenous Tianfu goose (*Anas cygnoides*) are commercially important farm animals in southern China. However, their poor egg-laying performance hinders their industrial application [16]. Many growing follicles undergo atresia as they advance toward ovulation. Numerous factors are involved in goose follicle maturation, and little attention has been paid to lipid metabolism in granulosa cells (GCs). In our previous research, we confirmed for the first time that de novo lipogenesis occurs in goose GCs [17]. Furthermore, *SCD* overexpression enhanced the intracellular level of cholesterol esters, particularly CE (20:4), which facilitates the synthesis and subsequent esterification of cholesterol into LDs in goose GCs [18]. While studies have demonstrated that *SCD* is an important rate-limiting enzyme in lipid metabolism, research on goose follicular steroidogenesis is lacking. Therefore, this study explored the effect of *SCD* on cell proliferation as well as total cholesterol (TC), estrogen, and progesterone levels in Tianfu goose GCs. Thus, we aim to deepen our understanding of the mechanisms of *SCD* in lipid metabolism during goose GC follicle development.

## 2. Materials and Methods

### 2.1. Animals and Granulosa Layer Isolation

A maternal line of Tianfu geese was raised under natural temperature and light conditions at the waterfowl breeding experimental station at Sichuan Agricultural University. For granulosa layer isolation, six geese showing regular laying schedules were randomly selected and sacrificed 2 h after oviposition via post-anesthesia exsanguination. A pool of hierarchical follicles was immediately collected from six goose abdominal cavities and placed in sterile normal saline. The outer connective tissue was removed from the follicles, and follicles were bisected to allow the yolk and adherent granulosa layer to flow out. GCs were isolated as previously described [19]. All experimental procedures involving animal manipulation were approved by the Committee of the School of Farm Animal Genetic Resources Exploration and Innovation Key Laboratory, College of Animal Science and Technology, Sichuan Agricultural University, under permit no. DKY20170913 and were performed in accordance with the Regulations of the Administration of Affairs Concerning Experimental Animals (China 1988). All efforts were made to minimize animal suffering in accordance with the requirements of the Beijing Animal Welfare Committee.

### 2.2. Primary Cell Culture and Transfection

The granulosa layer was dispersed by incubation in 0.1% type II collagenase (Sigma-Aldrich, Burlington, MA, USA) for 10 min in a 37 °C water bath. After centrifugation, the GCs were dispersed and pelleted by centrifugation at 1000× *g* for 10 min. Then, the supernatant was discarded, and GCs were resuspended in 3 mL of fresh basic medium without collagenase and centrifuged. The washing procedure was repeated twice. The GCs were dispersed in DMEM supplemented with 1% antibiotic/antimycotic solution (Solarbio, Beijing, China) and 3% fetal bovine serum (Gibco, Waltham, MA, USA). The viability of GCs was always greater than 90% and incubated in a water-saturated atmosphere of 95% air and 5% CO_2_ at 37 °C in an incubator (Thermo, Waltham, MA, USA). Transient transfections based on the GCs cellular model of *SCD* function (*SCD*-specific overexpression and knockdown) were performed using Lipofectamine^®^ 3000 and Lipofectamine™ RNAiMAX Transfection Reagent (Invitrogen, Waltham, MA, USA), according to the manufacturer’s protocol. *SCD*-specific overexpression was used to achieve *SCD* mRNA overexpression, termed pEGFP-N1/*SCD*, and the pEGFP-N1/empty vector (GFP vector) served as the negative control. Another control with no transfection components was also included. Specific small interfering RNA (*SCD* siRNA-210 and siRNA-405) were used to achieve *SCD* mRNA knockdown, with scrambled siRNA as a negative control. The primers of pEGFP-N1/*SCD* and the sequences of the siRNAs were determined as previously described [18,20].

### 2.3. Cell Proliferation Assay

The effect of *SCD* overexpression and *SCD* knockdown on the cell cycle stages of GCs was analyzed using an Accuri C6 flow cytometer (BD Biosciences, San Jose, CA, USA) with PI/RNase staining buffer (BD Biosciences). Briefly, 1 × 10^5^ cells were incubated overnight in 6-well plates. After 48 h transfection treatment, the cells were washed once with phosphate-buffered saline (PBS) solution and collected by centrifugation at 250× *g* for 5 min. Cells were then resuspended in 75% cold ethanol at −20 °C for 24 h. Before flow cytometry analysis, cells were washed with PBS and mixed with PI/RNase staining buffer for 30 min. Finally, the data were analyzed using ModFit software (Ashland, OR, USA). The cell proliferative index (PI) was calculated according to the following formula: PI (%) = (S + G2/M)/(G0/G + S+G2/M) × 100%, as described previously [21].

### 2.4. Measuring TC, Progesterone, and Estrogen Levels

After *SCD* overexpression and *SCD* knockdown in GCs, changes in the intracellular and extracellular cholesterol, estrogen, and progesterone levels were measured using the Goose TC ELISA Kit, Goose Progesterone ELISA Kit, and Goose estrogen (E) ELISA Kit (NJJCBIO, Nanjing, China), respectively. Each cell and culture medium was diluted five times with sample diluent, and 50 µL of the resultant dilution was added to the enzyme label plate. Plates were incubated at 37 °C for 30 min, washed five times with wash buffer, and air-dried at room temperature. A standard reagent (50 µL) was added to the plates, which were then washed five times. Next, 50 µL each of reagents A and B were added to the plates, which were incubated at 37 °C in the dark for 10 min. Finally, a 50 µL stop buffer was added, and the optical density (OD) value was measured using an automatic enzyme immunoassay analyzer at 450 nm.

### 2.5. Statistical Analysis

The experiment was repeated three times, and multiple means were compared using independent *t*-tests in the SPSS 19.0 statistical software (SPSS Inc., Chicago, IL, USA). All data are expressed as mean ± SD, and significance was assumed at *p* < 0.05. All data were visualized using GraphPad Prism 6.01 (GraphPad Software, San Diego, CA, USA).

## 3. Results

### 3.1. Effect of SCD Regulated Cell Cycle and Proliferation of Goose GCs

**Figure 1:**
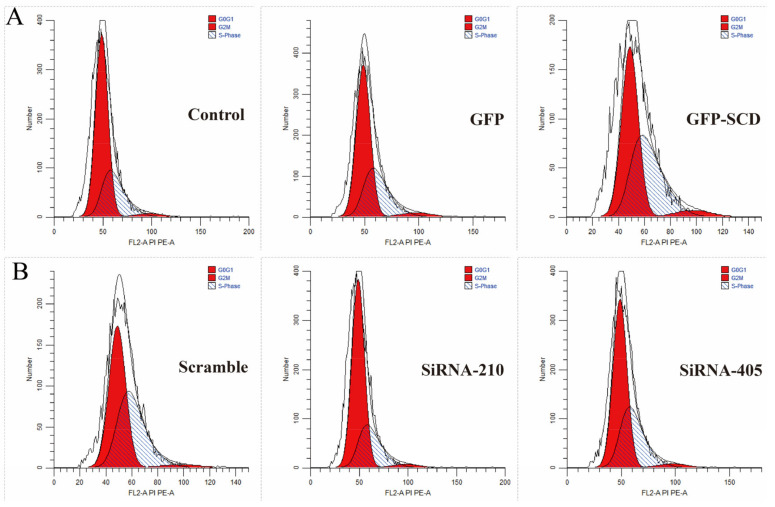
Effects of stearoyl-CoA desaturase (*SCD*) overexpression and *SCD* knockdown on the cell cycle of goose granulosa cells (GCs). (**A**) Cell cycle stages of GCs were analyzed after *SCD* overexpression. (**B**) GCs cell cycle stages were analyzed after *SCD* knockdown.

We used PI/RNase staining buffer to assess the goose GCs cell cycle after *SCD* overexpression and *SCD* knockdown (Figure 1). The results showed that after *SCD* overexpression, the proportion of GCs in the S phase was lower. The PI of GCs in the *SCD* overexpression group was markedly higher than that of the control or GFP groups (Table 1). We further conducted *SCD* knockdown studies as shown in Table 1. Compared with the scramble group, the S cells to PI ratio markedly decreased in both the siRNA-210 and siRNA-405 groups.

### 3.2. SCD Regulated TC Content of Goose GCs

**Figure 2:**
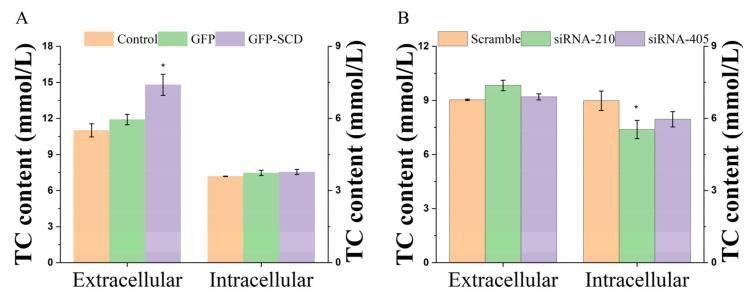
Changes in intracellular and extracellular total cholesterol (TC) content after transfection (Unit: mmol/L). (**A**) Overexpression-transfected group. (**B**) Small interfering RNA (siRNA)-transfected group. Asterisks indicate a significant intracellular or extracellular difference between groups (*p* < 0.05).

The production of TC content in the supernatant culture medium and cells was determined using ELISA. *SCD* overexpression led to significantly (*p* < 0.05) increased TC content in the culture medium. Additionally, the *SCD* overexpression group had higher TC content than the control and GFP groups (Figure 2A). In addition, compared with the scramble group, the TC content in the culture medium showed a post-transfection decrease in the siRNA-405 group; however, the siRNA-405 group revealed a contrasting trend. Meanwhile, *SCD* knockdown decreased TC content in the cells, among which the siRNA-210 group showed significantly (*p* < 0.05) decreased TC content in the cells (Figure 2B).

### 3.3. SCD Regulated Progesterone and Estrogen Secretion of Goose GCs

**Figure 3:**
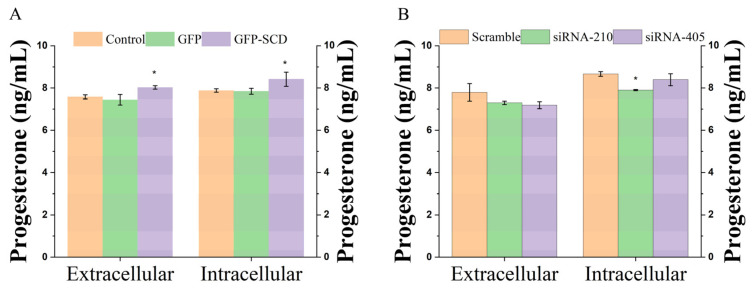

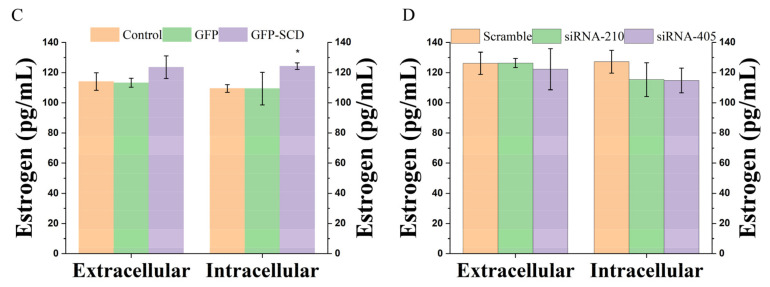
Changes in intracellular and extracellular progesterone and estrogen levels after transfection. (**A**,**B**) Change in progesterone (Unit: ng/L). (**C**,**D**) Change in estrogen (Unit: pg/L). Asterisks indicate a significant intracellular or extracellular difference between groups (*p* < 0.05).

We also investigated the effects of *SCD* overexpression and *SCD* knockdown on progesterone and estrogen secretion in goose GCs using ELISA. Compared with the control and GFP groups, *SCD* overexpression significantly (*p* < 0.05) increased progesterone secretion in the culture medium and cells (Figure 3A). Meanwhile, compared with the scramble group, progesterone secretion in the culture medium and cells decreased after *SCD* knockdown; among them, the siRNA-210 group significantly (*p* < 0.05) decreased progesterone secretion in the cells (Figure 3B). In addition, *SCD* overexpression increased estrogen secretion in the culture medium and significantly increased estrogen secretion in cells (Figure 3C). *SCD* knockdown decreased estrogen secretion in the culture medium and cells (Figure 3D).

## 4. Discussion

In the present study, we used a GC model of *SCD* function to obtain new evidence that *SCD* participates in GC proliferation and steroidogenesis. We demonstrated that *SCD* overexpression induced GC proliferation, whereas *SCD* knockdown inhibited GC proliferation (Table 1). *SCD* is the rate-limiting enzyme in the biosynthesis of monounsaturated fatty acids from saturated fatty acids, and accumulating evidence suggests *SCD* is a target for growth factors related to cell growth [22,23]. Notably, most cancer cells contain a high *SCD* activity to demand an even higher particular lipogenesis to support the rapid growth and de novo synthesis of membranes [8,24]. In our previous report, we investigated the metabolic mechanism of *SCD* during goose follicular development, among lipid metabolism-related pathways, LC-MS/MS analysis revealed the critical role of the steroid hormone biosynthesis/steroid biosynthesis pathway [20]. In this study, we confirmed that *SCD* overexpression increases TC content, and *SCD* knockdown decreases TC content in goose GCs, as evidenced by ELISA (Figure 2). The presence of normal MUFA levels would maintain a more appropriate ratio of cholesterol to other lipids, helping maintain cell membrane integrity [25]. Excess-free cholesterol has been known to lead to cell death [26]. It is tempting to speculate that TC content in the supernatant culture medium was higher than that in the cells, probably due to an increase in the amount of cellular free cholesterol secreted by cells. In addition, *SCD* is a resident ER protein; recent data show that an excess of SFAs and cholesterol disrupts lipid homeostasis in the ER membrane and induces ER stress, resulting in cell apoptosis and death [27]. Overall, these studies suggest that *SCD* activity and the availability of MUFAs correlate with malignant cell survival and proliferation [28,29].

Increasing evidence has shown the importance of lipid metabolism in goose follicle development [30,31]; we also demonstrated that de novo lipogenesis occurs in goose GCs [17], revealing that the LD accumulation capacity of goose GCs depends on the different stages of follicle development, with the highest accumulation capacity during the preovulatory follicle stage [32]. The accumulation of LDs in the cytoplasm plays a key role in the energy balance, membrane synthesis, and cell signal transduction [33,34]. In addition, *SCD* also plays a crucial role in lipid metabolism [35,36]; however, a causal relationship between the function of *SCD* and goose follicle development has not yet been well established. Further lipidomic profiling research on the LD accumulation capacity and abundance regulated by *SCD* in goose GCs identified that altered *SCD* expression affects LD content and changes lipid classes [18]. We speculate that endogenous LDs are a source of energy for steroidogenesis and proliferation in GCs and confirmed that *SCD* overexpression increases progesterone and estrogen secretion. Conversely, *SCD* knockdown decreases progesterone and estrogen secretion in goose GCs (Figure 3). Most other types of steroidogenic cells, such as those of the adrenal cortex, testes, and ovaries, contain tiny LDs that primarily store cholesterol esters and use them as substrates for steroid hormone synthesis [37,38]. We also confirmed that *SCD* overexpression results in increased esterification of cholesterol in goose GCs [20]. Similar studies have been confirmed in mice Chinese hamster ovary cells [39], whose results demonstrate that *SCD* expression is required to generate MUFAs as the preferred substrates for cholesterol ester synthesis.

**Figure 4:**
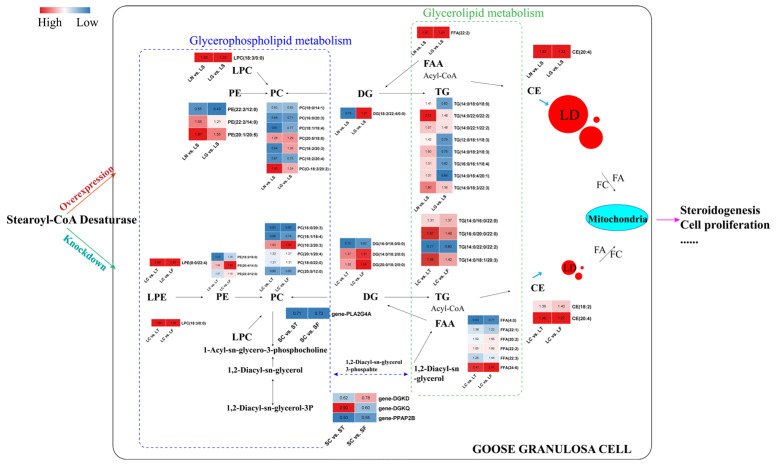
Diagram of stearoyl-CoA desaturase (*SCD*) regulation of key lipids and differentially expressed genes (DEGs) in glycerophospholipid metabolism and glycerolipid metabolism, which participate in steroidogenesis mediated by the deposition of lipid droplets in goose granulosa cells (GCs). Numbers in the heat map indicate the FC of the key lipids and DEG in the group; the color scale indicates the difference in FC; red, higher; blue, lower.

Although goose GCs store neutral lipids in LDs, there is little information on the molecular processes governing the deposition or catabolism of stored lipid components. Evidence indicates that *SCD* might be the pivotal control point regulating hepatic lipogenesis and lipid oxidation. *SCD* deficiency decreases the transcription of lipogenic genes, down-regulates de novo fatty acid synthesis, and increases the β-oxidation rate [40]. Consistent results were also confirmed in adipose tissues [41]. *SCD* deficient mice are characterized by a reduction in the percentage of fatty acids comprising MUFA, thereby significantly decreasing the synthesis of neutral lipids, such as triglycerides and cholesterol esters [39,42]. Even when the diet was supplemented with high levels of MUFA, decreasing *SCD* activity seemingly decreased lipid accumulation by limiting the supply of endogenously synthesized MUFAs [43]. There is ample evidence indicating that *SCD* can influence the partitioning of fatty acids into and out of neutral lipid species, which is essential in lipid metabolism and body weight control [44,45]. *SCD* is emerging as a potential therapeutic target for treating obesity, diabetes, and other metabolic diseases [46,47]. Lipid metabolism is highly regulated and complex; therefore, previous studies applied a multi-omics approach to analyze the changes in specific genes, metabolites, and lipid classes associated with *SCD* overexpression and *SCD* knockdown in goose GCs [18,20]. Combined with these studies, Figure 4 illustrates a potential mechanism of *SCD*-mediated regulation of key lipids as well as DEG of glycerophospholipid and glycerolipid metabolic pathways, participating in steroidogenesis and proliferation mediated by LD deposition in goose GCs.

## 5. Conclusions

In summary, our findings point to the vital role of *SCD* expression in mediating proliferation and steroidogenesis in goose GCs. These understandings should shed light on providing a new direction for exploring the mechanisms of lipid metabolism in goose follicle development.

## Figures and Tables

**Table 1:** Cell cycle results detected by flow cytometry.

Group	G0/G1 (%)	G2/M (%)	S (%)	PI (%)
Control	67.21	2.15	29.64	0.32
GFP	62.41	3.02	34.57	0.38
GFP-*SCD*	51.49	3.74	44.76	0.49
Scramble	52.51	1.91	45.58	0.48
SiRNA-210	69.99	2.28	27.73	0.30
SiRNA-405	60.99	2.44	36.57	0.39

## References

[1] Cohen P., Ntambi J.M., Friedman J.M. (2003). Stearoyl-CoA desaturase-1 and the metabolic syndrome. Curr. Drug Targets-Immune Endocr. Metab. Disord..

[2] Ntambi J.M., Miyazaki M. (2003). Recent insights into stearoyl-CoA desaturase-1. Curr. Opin. Lipidol..

[3] Ntambi J.M. (1995). The regulation of stearoyl-CoA desaturase (SCD). Prog. Lipid Res..

[4] Bell J.G., Tocher D.R., MacDonald F.M., Sargent J.R. (1995). Diets rich in eicosapentaenoic acid and γ-linolenic acid affect phospholipid fatty acid composition and production of prostaglandins E1, E2 and E3 in turbot (Scophthalmus maximus), a species deficient in Δ5 fatty acid desaturase. Prostaglandins Leukot. Essent. Fat. Acids.

[5] Miyazaki M., Gomez F.E., Ntambi J.M. (2002). Lack of stearoyl-CoA desaturase-1 function induces a palmitoyl-CoA Delta6 desaturase and represses the stearoyl-CoA desaturase-3 gene in the preputial glands of the mouse. J. Lipid Res..

[6] Paton C.M., Ntambi J.M. (2009). Biochemical and physiological function of stearoyl-CoA desaturase. Am. J. Physiol.-Endocrinol. Metab..

[7] Miyazaki M., Kim H.-J., Man W.C., Ntambi J.M. (2001). Oleoyl-CoA is the major de novo product of stearoyl-CoA desaturase 1 gene isoform and substrate for the biosynthesis of the harderian gland 1-Alkyl-2,3-diacylglycerol. J. Biol. Chem..

[8] Ntambi J.M. (1999). Regulation of stearoyl-CoA desaturase by polyunsaturated fatty acids and cholesterol. J. Lipid Res..

[9] Listenberger L.L., Han X., Lewis S.E., Cases S., Farese R.V., Ory D.S., Schaffer J.E. (2003). Triglyceride accumulation protects against fatty acid-induced lipotoxicity. Proc. Natl. Acad. Sci. USA.

[10] Jeyakumar S.M., Vajreswari A. (2022). Stearoyl-CoA desaturase 1: A potential target for non-alcoholic fatty liver disease?-Perspective on emerging experimental evidence. World J. Hepatol..

[11] Walther T.C., Farese R.V. (2012). Lipid droplets and cellular lipid metabolism. Annu. Rev. Biochem..

[12] Ralhan I., Chang C.L., Lippincott-Schwartz J., Ioannou M.S. (2021). Lipid droplets in the nervous system. J. Cell Biol..

[13] Zhang H., Su F., Huang L., Li B., Yuan X., Luo M., Ge L. (2021). Effects of progesterone on the lipolysis of lipid droplets and prostaglandin E(2) synthesis in murine cervical epithelial cells. Reprod. Fertil. Dev..

[14] McEvoy T.G., Coull G.D., Broadbent P.J., Hutchinson J.S., Speake B.K. (2000). Fatty acid composition of lipids in immature cattle, pig and sheep oocytes with intact zona pellucida. J. Reprod. Fertil..

[15] Kraemer F.B. (2007). Adrenal cholesterol utilization. Mol. Cell. Endocrinol..

[16] Phillips R.W., Johnson R.G., Moyer R.T. (1948). China’s goose industry. World’s Poult. Sci. J..

[17] Wen R., Gan X., Hu S., Gao S., Deng Y., Qiu J., Sun W., Li L., Han C., Hu J. (2019). Evidence for the existence of de novo lipogenesis in goose granulosa cells. Poult. Sci..

[18] Yuan X., Hu S., Li L., Han C., Liu H., He H., Xia L., Hu J., Hu B., Ran M. (2021). Lipidomics profiling of goose granulosa cell model of stearoyl-CoA desaturase function identifies a pattern of lipid droplets associated with follicle development. Cell Biosci..

[19] Gilbert A.B., Evans A.J., Perry M.M., Davidson M.H. (1977). A method for separating the granulosa cells, the basal lamina and the theca of the preovulatory ovarian follicle of the domestic fowl (*Gallus domesticus*). J. Reprod. Fertil..

[20] Yuan X., Hu S., Li L., Liu H., He H., Wang J. (2020). Metabolomic analysis of SCD during goose follicular development: Implications for lipid metabolism. Genes.

[21] Seifer D.B., Charland C., Berlinsky D., Penzias A.S., Haning R.V., Naftolin F., Barker B.E. (1993). Proliferative index of human luteinized granulosa cells varies as a function of ovarian reserve. Am. J. Obstet. Gynecol..

[22] Miyazaki M., Ntambi J.M. (2008). Role of stearoyl-coenzyme A desaturase in lipid metabolism. Prostaglandins Leukot. Essent. Fat. Acids.

[23] Mziaut H., Korza G., Ozols J. (2000). The N terminus of microsomal delta 9 stearoyl-CoA desaturase contains the sequence determinant for its rapid degradation. Proc. Natl. Acad. Sci. USA.

[24] Raeisi M., Hassanbeigi L., Khalili F., Kharrati-Shishavan H., Yousefi M., Mehdizadeh A. (2022). Stearoyl-CoA desaturase 1 as a therapeutic target for cancer: A focus on hepatocellular carcinoma. Mol. Biol. Rep..

[25] Garg M.L., Wierzbicki A.A., Thomson A.B., Clandinin M.T. (1988). Dietary cholesterol and/or n− 3 fatty acid modulate Δ9-desaturase activity in rat liver microsomes. Biochim. Biophys. Acta (BBA)-Lipids Lipid Metab..

[26] Tabas I. (2002). Consequences of cellular cholesterol accumulation: Basic concepts and physiological implications. J. Clin. Investig..

[27] Lai E., Bikopoulos G., Wheeler M.B., Rozakisadcock M., Volchuk A. (2008). Differential activation of ER stress and apoptosis in response to chronically elevated free fatty acids in pancreatic β-cells. Am. J. Physiol.-Endocrinol. Metab..

[28] Scaglia N., Caviglia J.M., Igal R.A. (2005). High stearoyl-CoA desaturase protein and activity levels in simian virus 40 transformed-human lung fibroblasts. Biochim. Biophys. Acta (BBA)-Mol. Cell Biol. Lipids.

[29] Morgan-Lappe S.E., Tucker L.A., Huang X., Zhang Q., Sarthy A.V., Zakula D., Vernetti L., Schurdak M., Wang J., Fesik S.W. (2007). Identification of ras-related nuclear protein, targeting protein for xenopus kinesin-like protein 2, and stearoyl-CoA desaturase 1 as promising cancer targets from an RNAi-based screen. Cancer Res..

[30] Li Q., Hu S., Wang Y., Deng Y., Yang S., Hu J., Li L., Wang J. (2019). mRNA and miRNA transcriptome profiling of granulosa and theca layers from geese ovarian follicles reveals the crucial pathways and interaction networks for regulation of follicle selection. Front. Genet..

[31] Chen X., Huang K., Hu S., Lan G., Gan X., Gao S., Deng Y., Hu J., Li L., Hu B. (2020). FASN-mediated lipid metabolism regulates goose granulosa cells apoptosis and steroidogenesis. Front. Physiol..

[32] Gao S., Gan X., He H., Hu S., Deng Y., Chen X., Li L., Hu J., Li L., Wang J. (2019). Dynamic characteristics of lipid metabolism in cultured granulosa cells from geese follicles at different developmental stages. Biosci. Rep..

[33] Benador I.Y., Veliova M., Liesa M., Shirihai O.S. (2019). Mitochondria bound to lipid droplets: Where mitochondrial dynamics regulate lipid storage and utilization. Cell Metab..

[34] Welte M.A., Gould A.P. (2017). Lipid droplet functions beyond energy storage. Biochim. Biophys. Acta (BBA)-Mol. Cell Biol. Lipids.

[35] Sun Y., Hao M., Luo Y., Liang C.P., Silver D.L., Cheng C., Maxfield F.R., Tall A.R. (2003). Stearoyl-CoA desaturase inhibits ATP-binding cassette transporter A1-mediated cholesterol efflux and modulates membrane domain structure. J. Biol. Chem..

[36] Ntambi J.M., Miyazaki M. (2004). Regulation of stearoyl-CoA desaturases and role in metabolism. Prog. Lipid Res..

[37] Plewes M.R., Krause C., Talbott H.A., Przygrodzka E., Wood J.R., Cupp A.S., Davis J.S. (2020). Trafficking of cholesterol from lipid droplets to mitochondria in bovine luteal cells: Acute control of progesterone synthesis. FASEB J..

[38] Talbott H.A., Plewes M.R., Krause C., Hou X., Zhang P., Rizzo W.B., Wood J.R., Cupp A.S., Davis J.S. (2020). Formation and characterization of lipid droplets of the bovine corpus luteum. Sci. Rep..

[39] Miyazaki M., Kim Y.C., Gray-Keller M.P., Attie A.D., Ntambi J.M. (2000). The biosynthesis of hepatic cholesterol esters and triglycerides is impaired in mice with a disruption of the gene for stearoyl-CoA desaturase 1. J. Biol. Chem..

[40] Ntambi J.M., Makoto M., Stoehr J.P., Hong L., Kendziorski C.M., Yandell B.S., Yang S., Paul C., Friedman J.M., Attie A.D. (2002). Loss of stearoyl-CoA desaturase-1 function protects mice against adiposity. Proc. Natl. Acad. Sci. USA.

[41] Lee S.H., Dobrzyn A., Dobrzyn P., Rahman S.M., Ntambi J.M. (2004). Lack of stearoyl-CoA desaturase 1 upregulates basal thermogenesis but causes hypothermia in a cold environment. J. Lipid Res..

[42] Miyazaki M., Man W.C., Ntambi J.M. (2001). Targeted disruption of stearoyl-CoA desaturase1 gene in mice causes atrophy of sebaceous and meibomian glands and depletion of wax esters in the eyelid. J. Nutr..

[43] Miyazaki M., Kim Y.C., Ntambi J.M. (2001). A lipogenic diet in mice with a disruption of the stearoyl-CoA desaturase 1 gene reveals a stringent requirement of endogenous monounsaturated fatty acids for triglyceride synthesis. J. Lipid Res..

[44] Dobrzyn A., Ntambi J.M. (2004). The role of stearoyl-CoA desaturase in body weight regulation. Trends Cardiovasc. Med..

[45] Tian H., Niu H., Luo J., Yao W., Chen X., Wu J., Geng Y., Gao W., Lei A., Gao Z. (2022). Knockout of stearoyl-CoA desaturase 1 decreased milk fat and unsaturated fatty acid contents of the goat model generated by CRISPR/Cas9. J. Agric. Food Chem..

[46] Shoelson S.E., Lee J., Goldfine A.B. (2006). Inflammation and insulin resistance. J. Clin. Investig..

[47] Dobrzyn A., Dobrzyn P., Lee S.H., Miyazaki M., Cohen P., Asilmaz E., Hardie D.G., Friedman J.M., Ntambi J.M. (2005). Stearoyl-CoA desaturase-1 deficiency reduces ceramide synthesis by downregulating serine palmitoyltransferase and increasing beta-oxidation in skeletal muscle. Am. J. Physiol.-Endocrinol. Metab..

